# Treatment Outcomes in Elderly with Advanced-Stage Non-small Cell Lung Cancer

**DOI:** 10.1007/s00408-013-9498-9

**Published:** 2013-08-09

**Authors:** Terence Chi-Chun Tam, James Chung-Man Ho, Matthew King-Yan Wong, Wai-Mui Wong, Julie Kwan-Ling Wang, Jamie Chung-Mei Lam, Macy Mei-Sze Lui, Wah-Kit Lam, Mary Sau-Man Ip, David Chi-Leung Lam

**Affiliations:** Department of Medicine, Queen Mary Hospital, University of Hong Kong, 102 Pokfulam Road, Hong Kong, HKSAR, China

**Keywords:** Lung cancer, Elderly, Treatment, Outcome

## Abstract

**Purpose:**

Lung cancer remains the top cause of cancer morbidity and mortality in the world. Although the identification of epidermal growth factor receptor (*EGFR*) gene mutations could predict efficacy of tyrosine kinase inhibitor (TKI), testing for predictive biomarkers are not always possible due to tissue availability. The overall therapeutic decision remains a clinical one for a significant proportion of elderly patients with advanced stage lung cancer but no known *EGFR* mutation status. The purpose of this study was to compare the outcome of drug treatment modalities in progression-free survival (PFS) and overall survival (OS) for elderly with advanced-stage non-small cell lung cancer (NSCLC) and to identify clinical parameters that could predict treatment outcome.

**Methods:**

Clinical records of patients aged 70 years or older with advanced-stage NSCLC who have received treatment were reviewed. A group of gender- and histology-matched subjects younger than age 70 years were identified as controls.

**Results:**

Fifty-six elderly patients were included. The median age at diagnosis was 73 years; 60.7 % received only one line of treatment. Baseline performance status (PS) was the only predictor of improved PFS (*p* = 0.042) and OS (*p* = 0.002). There was no difference in survival between the upfront chemotherapy and the TKI groups

**Conclusions:**

In elderly with advanced-stage NSCLC without known *EGFR* mutation status, use of *EGFR*–TKI and chemotherapy resulted in comparable survival benefits. Age was not predictive of worse treatment outcome. The baseline PS should be taken into consideration in the therapeutic decision in elderly with NSCLC where the EGFR mutation status is not known.

## Introduction

Lung cancer remains the top cause of cancer morbidity and mortality worldwide [1]. In Hong Kong, lung cancer ranks the top in cancer incidence and mortality [2]. In 2009, 4,365 new cases of lung cancer were diagnosed and this may be the most conservative estimation as underdiagnosis could occur in the frail and elderly [3], who may decline invasive investigation for histological diagnosis.

With increasing life expectancy over the past decades, the incidence of lung cancer in the elderly population is increasing. Approximately 50 % of new lung cancer was diagnosed in patients older than 65 years, while 30–40 % were diagnosed in patients older than 70 years [4]. In Hong Kong, the median age at diagnosis was 71 for men and 73 for women [2]. This subgroup does not only represent a significant healthcare burden, but their proportion is expected to grow with our aging population. However, elderly patients were underrepresented in clinical trials on cancer treatment (22 % of subjects were older than age 65 years, and 8–13 % older than age 70 years) [5]; the proportion of elderly (i.e., >70 years of age) included in the IDEAL 1 and 2 [6], INTACT [7, 8], IPASS [9], and NCIC–BR21 [10] studies were 4.9, 7.5, 27, and 22 % respectively, and the mean age of subjects in the TALENT [11] and TRIBUTE [12] trials were 59.1 ± 10.01 and 62.6 ± 10.1 years respectively. The results from these clinical trials might not be directly applicable to the elderly, as there could be diverse and variable effects of aging on organ functions. The variety of possible comorbid diseases also could result in heterogeneous therapeutic response in the elderly that is different from those seen in younger subjects. With the relative paucity of clinical trial data, the likelihood of receiving any kind of treatment for NSCLC, particularly chemotherapy, decreases significantly with increasing age [13]. Pharmacokinetic differences also may result in considerable variability in the efficacy and safety of cancer treatments in the elderly compared with the younger group of lung cancer subjects [14].

There were usually more female patients and adenocarcinoma in the younger NSCLC subjects compared with their older counterparts, but there was no statistical difference in the staging of lung cancer between the two age groups [15]. Data from five large clinical trials [SWOG 9509 [16], ECOG 5592 [17], ECOG 1594 [18], CALGB 9730 [19], and ECOG 4599 [20] trial] showed no difference between the young and old NSCLC patients in terms of the overall response rate and survival to chemotherapy treatment, but toxicity was slightly more prominent in the elderly. The ACCP Evidence-Based Clinical Practice Guidelines (2nd Edition, 2007) recommended that age alone should not dictate treatment-related decisions in patients with advanced NSCLC [21]. This recommendation was based largely on studies comparing younger (<70 years) with older (≥70 years) patients who participated in large, randomized trials that were not designed to address the elderly issue. An OS of 10.9 months was reported with the use of erlotinib [22]. In two other studies, survival and ORR were similar between gefitinib and vinorelbine [23] but were comparatively higher with erlotinib.

Chen et al. [24] found that in Chinese patients with advanced NSCLC aged 80 years or older, patients who received *EGFR–*TKI therapy had a significantly better prognosis (hazard ratio: 0.56), a benefit not found in chemotherapy or radiotherapy group. Irisa et al. [25] found that NSCLC histology (HR 1.631), three or more comorbidities (HR 1.317), and a CCI of more than three (HR 1.321) were independent negative prognostic factors. Girones et al. [26], however, found that TMN clinical staging (log-rank: *p* < 0.001), not CCI, was related to survival. Li et al. [27] found that comorbidity, number of chemotherapy cycles, and use of second-line therapy were identified as independent prognostic factors (Table 1).

**Table 1:** Summary of prognostic factors in elderly with advanced stage NSCLC

Author	Sample size (*n*)	Design	Significant prognostic factors
Hickish et al. [28]	290	Cisplatin chemotherapy	Performance status, disease extent, pattern of metastasis
Kaneda et al. [29]	101	Single use of gefitinib	Female gender, good performance status, low level of smoking index
Clement et al. [30]	231	Compared age <65 with ≥65	Extent of disease, hemoglobin level, time to first progression, presence of comorbidities
Yang et al. [31]	399	Chemotherapy-naïve Compared elderly with nonelderly	Platinum-based first-line chemotherapy, performance status, ORR for first-line treatment, regimen of second-line chemotherapy
Chan et al. [24]	203	Aged >80 Include both TKI and chemotherapy	*EGFR*–TKI use
Irisa et al. [25]	162	Aged >70 Include both TKI and chemotherapy	NSCLC histology, number of comorbidities, Charlson comorbidity score
Girones et al. [26]	83	Aged >70 Untreated patient	TMN staging
Li et al. [27]	109	Age >70 Treated with chemotherapy	Number of comorbidities, number of chemotherapy cycles, use of second-line therapy

Although the identification of epidermal growth factor receptor (*EGFR*) gene mutations could predict clinical efficacy of tyrosine kinase inhibitor (TKI), these molecular test for predictive biomarkers are not always possible or available due to tissue availability or financial constraint. Moreover, no reliable treatment outcome predictors could be identified for *EGFR* wild-type patients. Elderly patients with good PS and no major comorbid conditions seemed to derive benefits from carboplatin-based chemotherapy. However, no study had thus far considered all the above factors in analysis, nor has the sequence of therapy ever considered. There was no study that looked specifically at the tolerability of chemotherapy and TKI in elderly.

The aims of this study were to review and compare the use of systemic chemotherapy and *EGFR*–TKI and the clinical outcomes (OS, PFS, and complication rates) for elderly with advanced-stage NSCLC but no known *EGFR* mutation status.

## Materials and Methods

### Study Design

This was a single-center, retrospective cohort study in selected elderly patients with unresectable advanced stage or metastatic NSCLC. Clinical patient records with ICD coding of 162.0–162.9, from patients aged 70 years or older with pathological diagnosis of advanced stage (i.e., ≥Stage IIIA) NSCLC, and who had received treatment in the Department of Medicine, Queen Mary Hospital, from 2003 to 2009, were included. Primary endpoints were PFS and OS. OS was defined as the time from diagnosis to death. PFS was defined as the time from commencement of treatment to the time of documented disease progression or death, whichever came first. Adverse events were graded using the National Cancer Institute Common Toxicity Criteria Expanded Common Toxicity Criteria (in JBR 10) and version 2.0 of the National Cancer Institute Common Toxicity Criteria toxicity scale (in BR 18). Subjects younger than age 70 years, matched for gender, histology, and smoking history in the same time period were identified as the control cohort, and the OS was compared.

### Clinical and Statistical Variables

The following variables were included in the analysis: gender, smoking history, drinking history, number of comorbidities, Charlson Comorbidity Index (CCI), Simplified Comorbidity Score (SCS), primary site of the tumor, location of metastasis, TMN stages, standardized uptake value (SUV) in positron-emission tomography (PET-CT), cell types, degree of differentiation reported in the histology report, types of first-line treatment received (*EGFR*–TKI vs. chemotherapy), and total numbers of lines of treatment. Survival and complications rate also were analyzed. Kaplan–Meier analysis was used to compare survival between those aged <70 and >70 years.

### Statistical Methods

The IBM PASW 17.0 was used for statistical analysis. Data were expressed as frequency, mean, standard deviation and range as appropriate. All tests were two-tailed, and statistical significance was *p* ≤ 0.05. χ^2^ test was used for categorical variables, and Fisher’s exact test was used for smaller group sizes (i.e., less than five in a group). Mann–Whitney test was employed for analysis of continuous variables. PFS and OS were assessed by Kaplan–Meier analysis. A proportional Cox regression model was applied to identify explanatory variables for survival and to calculate hazard ratio.

## Results

### Patient Demographics

A total of 1,998 lung cancer subjects with the diagnosis coding of 162.1–162.9 (neoplasm of lung) who fulfilled the inclusion criteria were retrieved from the Hong Kong Hospital Authority Clinical Management System; 1,500 subjects remained after small cell lung cancer, neuroendocrine tumor of the lung, and cancer due to metastasis were excluded. With the additional criteria of history of use of EGFR–TKI (gefitinib or erlotinib) or chemotherapy (including cisplatin, carboplatin, docetaxel, paclitaxel, gemcitabine, vinorelbine, pemetrexed, and etoposide), 300 patients remained. Fifty-six of these patients were aged ≥70 years. Of these 56 patients, the median age at the time of diagnosis was 73 years (interquartile range (IQR), 71.25–75.00 years). Nineteen (33.9 %) patients were nonsmokers, 27 (48.2 %) were former smokers, and 10 (17.9 %) were current smokers. Nineteen (33.9 %) patients enjoyed good past health before cancer diagnosis, and eight (14.3 %) of these patients had a prior history of cancer in other organ systems. CCI scores ranged from 0 to 8, whereas SCS ranged from 0 to 6. Four (7.1 %) had a family history of cancer. Thirty-eight (67.9 %) presented incidentally, and 49 (87.5 %) had PS of 0–1 at the time of diagnosis of lung cancer [compared with 7 (12.5 %) with PS 2–4]. Intrapulmonary metastasis was suspected or confirmed in 14 (25 %), brain metastasis in 3 (5.4 %), liver metastasis in 2 (3.6 %), adrenal metastasis in 9 (16.1 %), bone metastasis in 12 (21.4 %), pericardial effusion in 3 (5.4 %), and pleural effusion in 19 (34.5 %) of patients. Ten patients (17.9 %) had stage IIIA, 12 (21.4 %) with stage IIIB, and 34 (60.7 %) had stage IV disease. Twelve (21.4 %) had NSCLC, 31 (55.4 %) had AD, and 9 (16.1 %) had SCC. Thirty-four (60.7 %) patients received only one line of treatment (either TKI or chemotherapy), 4 (7.1 %) had TKI followed by chemotherapy on progression, and the remaining 18 (32.1 %) had chemotherapy followed by TKI on progression; 13 (23.3 %) had more than two successive lines of different therapy. The most commonly employed first line chemotherapy was paclitaxel–platinum doublets (44 %), followed by gemcitabine–platinum doublets (28.9 %), whereas monotherapy was used as first-line treatment in three patients (6.7 %; Table 2).

**Table 2:** Baseline demographics of included subjects

	Number of patients (%)
Gender	
Female	20 (35.7)
Male	36 (64.3)
Smoking history	
Non-smoker	19 (33.9)
Ex-smoker	27 (48.2)
Current smoker	10 (17.9)
Drinking history	
Non-drinker	46 (82.1)
Ever-drinker	10 (17.9)
Good past health	
No	37 (66.1)
Yes	19 (33.9)
Cancer history	
No	48 (85.7)
Other cancer	8 (14.3)
CCI score	
0	29 (51.8)
1	14 (25)
2	5 (8.9)
≥3	8 (14.3)
SCS score	
0	16 (28.6)
1	18 (32.1)
2	7 (12.5)
≥5	15 (25 %)
Family history of cancer	
No	52 (92.9)
Lung cancer	2 (3.6)
Other cancer	2 (3.6)
Incidental presentation	
No	38 (67.9)
Yes	18 (32.1)
PS	
0–1	49 (87.5)
2–4	7 (12.5)
Metastasis	
Intrapulmonary	
Suspected	5 (8.9)
Confirmed	9 (16.1)
Brain	
Confirmed	3 (5.4)
Liver	
Confirmed	2 (3.6)
Adrenal	
Suspected	4 (7.1)
Confirmed	5 (8.9)
Bone	
Suspected	1 (1.8)
Confirmed	11 (19.6)
Pericardial effusion	
Suspected	1 (1.8)
Confirmed	2 (3.6)
Pleural effusion	
Suspected	12 (21.8)
Confirmed	7 (12.7)
T-staging	
1A	3 (5.4)
2A	11 (19.6)
2B	4 (7.1)
T-staging	
3	12 (21.4)
4	22 (39.3)
NA	4 (7.1)
N-staging	
0	7 (12.5)
1	5 (8.9)
2	16 (28.6)
3	26 (46.4)
NA	2 (3.6)
M-staging	
0	22 (39.3)
1a	19 (33.9)
1b	15 (26.8)
Stage	
IIIA	10 (17.9)
IIIB	12 (21.4)
IV	34 (60.7)
Cell type	
AD	27 (48.2)
Non-AD	29 (51.8)
Treatment combination	
Single type	34 (60.7)
TKI then chemotherapy	4 (7.1)
Chemotherapy then TKI	18 (32.1)
Total lines of treatment	
1	27 (48.2)
2	16 (28.6)
≥3	13 (23.2)

### First-Line TKI Versus Chemotherapy

As shown in Table 3, there was no difference between the first-line TKI and chemotherapy group in terms of mean age (71 vs. 73 years; *p* = 0.193), smoking status, good past health or not, prior cancer history, CCI scores, SCS scores, family history of cancer and baseline performance status (PS), the location of the primary tumor, and the sites of metastasis and the overall TNM staging between the two groups. There also was no statistical difference between the two groups in terms of the ultimate numbers of lines of treatment received. The only statistically significant difference was found in those who received *EGFR–*TKI as first-line therapy were more likely to be female (male:female 64.3:35.7 % vs. 27.3:72.7 %; *p* = 0.011), and patients who had upfront chemotherapy were more likely to have received just one line of treatment (*p* < 0.001; Table 3).

**Table 3:** Comparison between upfront TKI and chemotherapy groups

	Number of patients (%)		*p*
	First-line: TKI	First-line: chemotherapy	
Gender			
Female	8 (72.7)	12 (26.7)	0.011*
Male	3 (27.3)	33 (73.3)	
Smoking history			
Non-smoker	5 (45.5)	14 (31.1)	0.564
Ex-smoker	5 (45.5)	22 (48.9)	
Current smoker	1 (9.1)	9 (20)	
Drinking history			
Non-drinker	11 (100)	35 (77.8)	0.226
Ever-drinker	0	10 (22.3)	
Good past health			
No	5 (45.5)	32 (71.1)	0.156
Yes	6 (54.5)	13 (28.9)	
Cancer history			
No	11 (100)	37 (82.2)	0.333
Other cancer	0	8 (17.8)	
CCI score			
0	8 (72.7)	21 (46.7)	0.849
1	2 (18.2)	12 (26.7)	
2	1 (9.1)	4 (8.9)	
≥3	0	8 (17.8)	
SCS score			
0	6 (54.5)	10 (22.2)	0.093
1	3 (27.3)	15 (33.3)	
2	0	7 (15.6)	
≥3	2 (18.2)	13 (28.9)	
Family history of cancer			
No	10 (90.9)	42 (93.3)	0.436
Lung cancer	0	2 (4.4)	
Other cancer	1 (9.1)	1 (2.2)	
Incidental presentation			
No	8 (72.7)	30 (66.7)	0.501
Yes	3 (27.3)	15 (33.3)	
PS			
0–1	9 (81.8)	40 (88.9)	0.614
2–4	2 (18.2)	5 (11.1)	
Metastasis			
Intrapulmonary			
Suspected	2 (18.2)	3 (6.7)	0.42
Confirmed	1 (9.1)	8 (17.8)	
Brain			
Confirmed	1 (9.1)	2 (4.4)	0.488
Liver			
Confirmed	0	2 (4.4)	0.654
Adrenal			
Suspected	1 (9.1)	3 (6.7)	0.961
Confirmed	1 (9.1)	4 (8.9)	
Bone			
Suspected	0	1 (2.2)	0.87
Confirmed	2 (18.2)	9 (20.0)	
Pericardial effusion			
Suspected	0	1 (2.2)	0.679
Confirmed	0	2 (4.4)	
Pleural effusion			
Suspected	3 (27.3)	9 (20.5)	0.684
Confirmed	2 (18.2)	5 (11.4)	
T-staging			
1A	0	3 (6.7)	0.533
2A	3 (27.3)	8 (17.8)	
2B	2 (18.2)	2 (4.4)	
T-staging			
3	2 (18.2)	10 (22.2)	
4	3 (27.3)	19 (42.2)	
NA	1 (9.1)	3 (6.7)	
N-staging			
0	3 (27.3)	4 (8.9)	0.147
1	0	5 (11.1)	
2	1 (9.1)	15 (33.3)	
3	6 (54.5)	20 (44.4)	
NA	1 (9.1)	1 (2.2)	
M-staging			
0	4 (36.4)	18 (40)	0.624
1a	5 (45.5)	14 (31.1)	
1b	2 (18.2)	13 (28.9)	
Stage			
IIIA	0	10 (22.2)	0.143
IIIB	4 (36.4)	8 (17.8)	
IV	7 (63.6)	27 (60)	
Cell type			
AD	6 (54.5)	21 (46.7)	0.639
Non-AD	5 (45.5)	24 (53.3)	
Treatment combination			
Single type	5 (45.5)	29 (64.4)	<0.001*
TKI then chemotherapy	4 (36.4)	0	
Chemotherapy then TKI	2 (18.2)	16 (35.6)	
Total lines of treatment			
1	4 (36.4)	23 (51.1)	0.072
2	3 (27.3)	13 (28.9)	
≥3	4 (36.4)	9 (20)	

* *p* < 0.05

### Survival Analysis

**Fig. 1:**
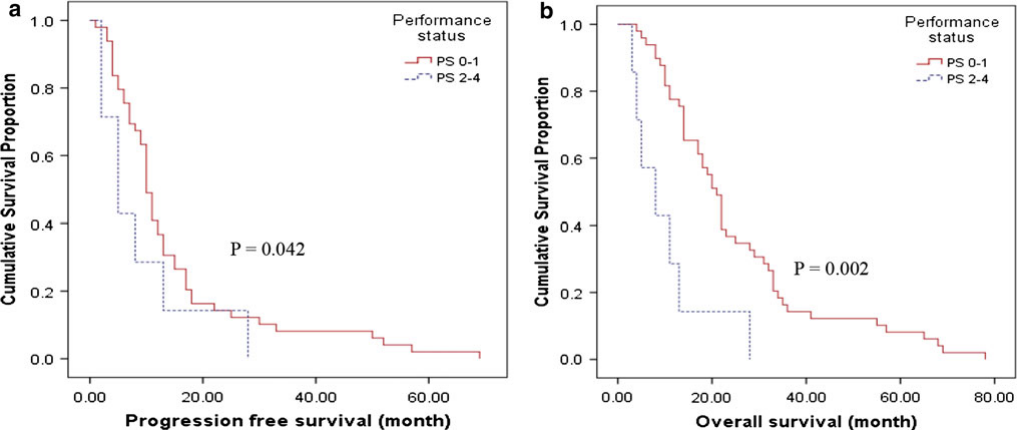
Kaplan–Meier curves for **a** PFS and **b** OS in relation to PS

Overall PFS and OS for the whole group of 56 patients were ten (range 5–15) months and 19 (range 11–31) months respectively. In univariate analysis, longer PFS correlated with better baseline PS (Fig. 1). Gender, smoking history, and cell type did not predict PFS. On the other hand, longer OS was correlated with better baseline PS, AD cell types, and increased lines of treatment and better CCI scores. Age, smoking history, drinking history, SCS, choice of upfront treatment (*EGFR*–TKI or chemotherapy) and whether patients had ever used TKI were not associated with survival.

### Complication Rates

The rates of severe adverse effects of chemotherapy and *EGFR*–TKI are summarized in Table 4. Fourteen of 51 (27.5 %) patients who have ever undergone chemotherapy had no complications, comparable to the eight of 27 (29.6 %) in ever-TKI group (Table 4). No factors (including age, PS, CCI, SCS, or cell type) were found to be predictive of treatment (both chemotherapy and TKI) intolerance.

**Table 4:** Comparison of adverse effects

	Chemotherapy (%)				TKI (%)			
Grade	1	2	3	4	1	2	3	4
Neutropenia	10.7	17.9	17.9	0	0	0	0	0
Renal impairment	7.1	0	5.4	0	9.0	0	0	0
Liver impairment	1.8	3.6	1.8	0	0	0	1.8	0
Neuropathy	3.6	0	1.8	0	0	0	0	0
Skin reaction	5.4	0	0	0	12.5	16.1	1.8	1.8
Pulmonary reaction	0	0	1.8	1.8	0	0	0	1.8
Others	0	0	0	1.8	9.0	3.6	0	1.8

### Subgroup Analysis

The records of 56 additional patients with advanced-stage NSCLC but younger than age 70 years matched for gender, histological subtypes, and smoking status to our elderly (age ≥70 years) cohort, were reviewed. The mean age of this group was 47 (range 40–53.75) years. The OS was 14 (range 8–29) months, which was not significantly different from their elderly counterparts (*p* = 0.075). Gender has no significant correlation with OS (*p* = 0.255 and *p* = 0.168 for female and male subgroups respectively).

## Discussion

### Clinical Significance

This study gave insights into local practice of lung cancer treatment for the elderly. The results served as a reminder of the importance of lung cancer in the elderly and their needs for special attention.

The survival predictors identified in this study were different from those published by Hickish et al. [28] (PS, extent of disease, pattern of metastasis, and age) and Kaneda et al. [29] (female gender, good PS, low level of smoking index). This could be the results of inclusion of both chemotherapy- and TKI-treated elderly based on clinical judgment. The study by Chen et al. [24] (survival predictor—use of TKI) were similar to our study but may not be directly comparable as patients aged 70–79 years were excluded. On the contrary, the studies by Irisa et al. [25] (survival predictor—histology subgroup, number of comorbidities, and CCI score) and Girones et al. [26] (survival predictor—TMN staging) shared the same age cutoff of 70 years and overall study design. We have considered all the variables mentioned in these studies, but none was found to be a significant survival predictor; one possible explanation of this might be a sample bias as those with multiple comorbidities might not have been referred for treatment. Our findings were most similar to those demonstrated by Li et al. [27] (survival predictors—comorbidity, chemotherapy cycles, and presence of second-line therapy). However, *EGFR*–TKI use was not included in those studies. To our knowledge, our study was the only one currently available that reviewed specifically Chinese elderly aged ≥70 years with advanced NSCLC who has undergone treatment in accordance with contemporary standard of care. In addition to verifying the previously published prognostic factors, our study was the first to look at whether inclusion of TKI in the treatment or the choice of first-line therapy affected survival.

Although *EGFR* mutation status could be tested for lung cancer patients to guide therapeutic decision for using *EGFR*–TKI or not, such biomarker testing may not always be possible or available due to tissue availability or financial constraint. In this retrospective review of treatment outcome in elderly patients with advanced-stage lung cancer (*EGFR* mutation testing was not available in our hospital service within the review period), PS was found to be the only significant determining factor for survival outcome in elderly subjects with advanced stage NSCLC. Neither age nor choice of upfront treatment (chemotherapy or TKI) was a significant predictor for survival. Tolerability of chemotherapy and TKI in the elderly was similar, and our subanalysis showed that the survival in the elderly were similar to that of their younger counterpart. These results suggested that the general PS of the patients, rather than age alone, should be one clinical parameter used to guide therapeutic decisions as to the choice between TKI and chemotherapy. The results of this retrospective study would pave the way for further prospective study on the treatment of advanced stage lung cancer in elderly subjects.

The performance of CCI and SCS as two potentially useful scores to guide treatment decision in the elderly was reviewed in this study. CCI was a significant prognostic factor to predict OS in univariate analysis, but not in multivariate analysis. This could partly be explained by the fact that CCI was designed for elderly, hospitalized patients, and therefore might not be informative in our NSCLC subsets who were managed as out-patient as far as was practically possible. SCS was not found to be a significant outcome predictor of survival. This difference from published data was likely due to the fact that the initial SCS derivation utilized patients from all stages of NSCLC, and the median age of that study was only 62.5 years.

Despite the common practice (at the time) of treating older patients with monotherapy, only three patients (6.7 %) in our cohort received first-line monotherapy, whereas all others received platinum-doublets. Our experience was that most doublet regimen are well tolerated in the elderly, and this echoed the finding in the IFCT-0501 trial in which patients aged at least 70 years were randomized to receive either vinorelbine or gemcitabine alone or with monthly carboplatin combined with weekly paclitaxel demonstrated that there was a highly significant benefit of survival in the doublet chemotherapy arm [30]. Even in a priori unfavourable prognostic subgroups (patients with a PS score of two, those aged >80 years or those with an activities of daily living scale score of <6), doublet therapy was associated with a survival advantage over monotherapy

The sample inclusion in this study spanned over 7 years, during which management protocols, recommendations, and even staging system have changed (most of our clinical management was based on the sixth edition of the UICC TNM Staging system and treatment suggestions). Some of these patients could have been managed differently nowadays, in keeping with the most updated seventh edition of the UICC/IASLC manual for lung cancer staging. An invariable bias in our study stems from its retrospective design. A cardinal example would be the preponderance of female patient in the EGFR–TKI subgroup (72.7 vs. 35.7 %), which is likely due to clinical selection bias on the part of the treating clinicians. In addition to physicians’ clinical decision on the choice of treatment, namely different types of chemotherapy or TKI, patient’s choices played an important role in the choice of treatment. Education levels (the adequacy to understand presented information and critical analysis ability), patient’s preference (which might be dictated by perception in adverse effect profile rather than efficacy), and social and financial factors might confound treatment selection. Our study allowed for all treatment as per protocol and practical factors, and therefore the results obtained can provide practical information for practicing medical practitioners and nursing staff.

In clinical practice, invasive procedures often were not considered for those that have poor premorbid state, based on the rationale that patients might not be able to tolerate any treatment even after invasive investigation. Therefore, the included subjects in this study population might have better pre-morbid state. *EGFR* mutation testing was not widely available at the time of this study, and none of the included patients have known *EGFR* mutation status. Despite the common dictum in the pre-*EGFR*-testing era to select nonsmoking women with adenocarcinoma for TKI therapy, our data failed to show obvious association of these features with clinical response to TKI. A possible reason was again patients’ choice, which depends heavily on their perceived side effect profile of the treatment. Elderly patients and their family might choose TKI since they believe that it is less toxic, and therefore more tolerable, than chemotherapy.

Our finding of similar survival between elderly and their younger counterparts echoed those published; data from five large clinical trials [SWOG 9509 [31], ECOG 5592 [32], ECOG 1594 [33], CALGB 9730 [34], and ECOG 4599 [35] trial] found no difference between the young and old NSCLC patients in terms of the overall response rate and survival to chemotherapy treatment, but toxicity was slightly more prominent in the elderly.

The survival (both PFS and OS) was significantly longer in our study compared with existing elderly-focused trials (e.g., ELVIS or MILES). However, the OPTIMAL trial has shown that median PFS reached 13.1 months in patients with EGFR-activating mutation treated with EGFR–TKI [36]. In the iPASS trial, it was shown that PFS ranges from 5.5–6.3 months in the chemotherapy arm [9]. Based on these two Asian-oriented trials, it can be postulated that the observed survival in this study might be due to a significant number of patient that have harbored EGFR-activating mutation.

Although the small sample size of this study preclude meaningful multivariate analysis, empirical data (not shown in the result session) seems to point to PS as the only significant predictive factor to PFS (HR 1.792, CI 1.022–3.143, *p* = 0.042) and OS (HR 1.921, CI 1.259–2.907, *p* = 0.002). To confirm this, along with other findings in this study, a similarly designed, prospective trial with larger sample size would be informative.

It is important and warranted to include more elderly patients in prospective clinical trials in the use of both chemotherapy and targeted therapeutics to better determine the factors that should aid decision to treat with chemotherapy or targeted therapies and to address the comparison between doublet and single-agent therapy.

## Conclusion

In elderly subjects with advanced-stage NSCLC without known *EGFR* mutation status, *EGFR*–TKI appeared non-inferior compared with chemotherapy. Age was not a significant predictor of outcome but PS before primary treatment could be one predictor of clinical outcome of treatment. Further, larger, prospective studies in elderly subjects with advanced stage NSCLC are needed to guide clinical management and therapeutic decision and to improve treatment outcomes of elderly subjects with advanced stage NSCLC.
